# The instantaneous helical axis of the subtalar and talocrural joints: a non-invasive *in vivo *dynamic study

**DOI:** 10.1186/1757-1146-3-13

**Published:** 2010-07-13

**Authors:** Frances T Sheehan

**Affiliations:** 1 Functional and Applied Biomechanics Section, Rehabilitation Medicine Department, National Institutes of Health, Bethesda, MD, USA

## Abstract

**Background:**

An understanding of rear-foot (talocrural and subtalar joints) kinematics is critical for diagnosing foot pathologies, designing total ankle implants, treating rear-foot injuries and quantifying gait abnormalities. The majority of kinematic data available have been acquired through static cadaver work or passive *in vivo *studies. The applicability of these data to dynamic *in vivo *situations remains unknown. Thus, the purpose of this study was to fully quantify subtalar, talocrural and calcaneal-tibial *in vivo *kinematics in terms of the instantaneous helical axis (IHA) in twenty-five healthy ankles during a volitional activity that simulated single-leg toe-raises with partial-weight support, requiring active muscle control.

**Methods:**

Subjects were each placed supine in a 1.5 T MRI and asked to repeat this simulated toe-raise while a full sagittal-cine-phase contrast (dynamic) MRI dataset was acquired. From the cine-phase contrast velocity a full kinematic description for each joint was derived.

**Results:**

Nearly all motion quantified at the calcaneal-tibial joint was attributable to the talocrural joint. The subtalar IHA orientation and position were highly variable; whereas, the talocrural IHA orientation and position were extremely consistent.

**Conclusion:**

The talocrural was well described by the IHA and could be modeled as a fixed-hinge joint, whereas the subtalar could not be.

## Background

An understanding of rear-foot kinematics is critical for diagnosing/treating foot pathologies and injuries [1-3], designing total ankle implants [4,5], and quantifying gait abnormalities. The complicated foot-ankle complex is composed of 26 bones that transfer ground reaction forces to the lower limb. Due to its role in transferring these forces to the rest of the body, the rear-foot is frequently exposed to injury and pathology. For example, ankle sprains account for roughly 25% of all sports related injuries, making it the most common sports-related injury [6]. Osteoarthritis secondary to trauma is also common [7]. Total ankle arthroplasty is often considered for end stage arthritis, but the long term success does not match that found for the proximal leg joints [8]. A common thread amongst these pathologies and injuries is that intervention would likely be enhanced with accurate *in vivo *rear-foot kinematic and kinetic data. Gougoulias and colleagues [7] stated, "The frequent failure of ankle implants may be related to .....poor reproduction of the normal mechanics of the ankle (talocrural) joint". Without knowledge of *in vivo *talocrural and subtalar motion during volitional exercise under active muscle control, "normal" mechanics cannot be understood and thus, cannot be reproduced. Therefore, implant design may be enhanced with *in vivo *data acquired during dynamic tasks requiring active muscle control.

The majority of kinematic data (Table 1) available for the tibial-talus (talocrural) and talus-calcaneus (subtalar) joints have been acquired through static cadaver work [9-13] or passive *in vivo *experiments [14-17]. In general, these studies presented data in terms of the finite helical axis (FHA), typically defined as the axis of rotation between two extreme static poses (e.g., extreme plantarflexion to extreme dorsiflexion). These studies have led to an overall assumption that rear-foot kinematics can be modeled by two fixed hinge joints [18-21]. Plantarflexion-dorsiflexion (PF-DF) is assumed to occur at the talocrural joint and inversion-eversion coupled with internal-external rotation is assumed to occur at the subtalar joint. Yet, cadaver-based experiments were unable to quantify the change in the FHA throughout a range of motion during a volitional task (a voluntary motion under active muscle control). Two studies did report the rotation about [22] and orientation of [23] the subtalar FHA during volitional dynamic activities, the former included data for the talocrural joint as well. Both studies used highly invasive bone screws, with a small number of subjects (n = 3 and n = 8). Thus, the bulk of the data available for rear-foot kinematics lack information in regards to *in vivo *joint motion during volitional activity. More importantly, no studies have provided a complete kinematic definition of the FHA for either joint. As described by Woltring and colleagues [24], the FHA is only fully defined when: the three-dimensional direction of the FHA (**n**), the three-dimensional location of a single point on the FHA (**s**), the rotation about the FHA (θ) and translation along the FHA are provided.

**Table 1:** Summary of Previous Rear-Foot FHA and IHA studies (in date order).

Talocrural							
**Study**	**Study type**	**#**	**Inclination**				

			**Cor**	**Sag**	**Ax**	**Rot**	**Trans**

Inman [11]	Static, cadaver (max PF - DF)	49	82° (SD 3.6°)	--	--	--	--

Manley [10]	Static, cadaver (max PF - DF)	--	80° (SD 10°)	--	84°	--	--

Lundberg [16]	Static,*in vivo* (max PF - DF)	8	--	--	-2° (SD 5°)	--	--

van den Bogert [21]	Model optimization with *in vivo *data	14	--	6.84°	19.1°	--	--

Arndt [23]	*in vivo*, gait (begin-end PF)	3	--	7.5° - 33.6°	56.1° -69.9°	--	--

Pearce [17]	Static,*in vivo* (max inv -ev)	20	--	--	--	5.2° (SD 2.2°)	--

Siegler [15]	Static,*in vivo* (neutral to inv)	7	--	--	--	8.4° (SD 5.7°)	3 mm (SD 2.5 mm)

**Current**	*in vivo*, simulated toe-raise	25	84.5° (SD 12.9°)	22.0° (SD 41.7°)	105.8° (SD 12.2°)	31.7° (SD 11.3°)	-0.5 mm (SD 1.4 mm)

**Subtalar**							

Manter [13]	Static cadaver (max PF - DF)	--	--	42° (range 29°-47°)	16 (range 8° -24°)	--	--

Root [9]	Static, cadaver (max PF - DF)	22	--	41° (SD 8.36°)	17° (SD 2.23°)	--	--

Close [22]	*in vivo*, gait	8				17.6° (SD 6.7°)	

Inman [11]	Static, cadaver (max PF - DF)	49	--	42° (SD 9°)	23 (SD 11°)	--	--

Manley [10]	Static, cadaver	--	--	41°	23°	--	--

Lundberg [16]	Static,*in vivo* (max PF - DF)	8	--	34° (SD 16°)	32° (SD 16°)	--	--

Pearce [17]	Static,*in vivo* (max inv -ev)	20	--	--	--	10.9° (SD 3.3°)	--

Leardini [12]	Static, cadaver (max Inv. - Ev)	6	--	range 43.5°-60.8°	range 32.8°-46.5°	--	--

Arndt [23]	*in vivo*, gait (begin-end PF)	2	--	31.4° - 36.45°	15.7° -23.5°	--	--

Siegler [15]	Static,*in vivo* (neutral to inverted)	7	--	--	--	9° (SD 4°)	1.9 mm (SD 1.2 mm)

Biemers [14]	Static,*in vivo* (max PF- DF)	20	--	9.5° (SD 46.8°)	23.6° (SD 30.1°)	7.3° (SD 6.0)	1.mm (SD 2.1 mm)

**Current**	*in vivo*, simulated toe-raise	25	variable	variable	variable	15.1° (SD 9.7°)	-0.3 mm (SD 1.4 mm)

The abbreviations used are: # - number of subjects or specimens; Cor - coronal plane; Sag - sagittal plane; Ax - axial plane; Rot - rotation about the FHA; Trans - translation along FHA and SD - standard deviation. "Max PF-DF", "max Inv-Ev", and "Neutral to inverted" indicate that the FHA was defined as the change in joint attitude between two poses (extreme PF to extreme DF, extreme Inversion to extreme Eversion, and neutral to extreme inversion respectively).

Thus, the purpose of this study was to fully quantify subtalar, talocrural and calcaneal-tibial joint kinematics in terms the Instantaneous Helical Axis (IHA), during an activity that simulated single-leg toe-raises with partial-weight support, requiring active muscle control, in healthy volunteers. The use of cine-PC MRI allowed the IHA to be calculated directly from the angular velocity, as this technique was able to quantify musculoskeletal velocities during a dynamic movement. This was in contrast to numerous previous studies that defined rear-foot kinematics using the FHA, calculated between two discrete positions. Although the calcaneal-tibial was not a true joint, it was included because it has been used to describe rear-foot motion when talar kinematics were not available. A secondary purpose was to determine the relative contributions of the subtalar and talocrural joints to calcaneal-tibial rotation, during a functional task requiring active muscle control.

## Methods

Twenty asymptomatic volunteers provided informed consent to participate in this Institutional Review Board-approved study. Subjects were excluded if they had any contraindications to magnetic resonance (MR) imaging, reported previous foot impairment, pathology, pain or surgery. For fifteen subjects, the side (right of left) studied was selected at random and for five subjects both rear-feet were studied, because scanning time permitted. Thus, in total, 25 rear-feet were included within the study (age = 26.2 ± 4.5 years; weight = 71.1 ± 13.3 kg; height = 173.6 ± 7.2 cm, 6F/19M).

**Figure 1:**
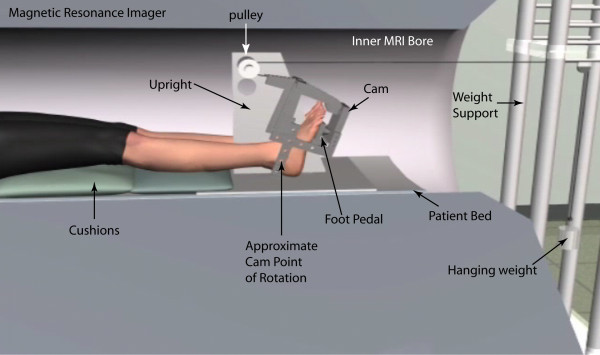
**Subject placement within the MR imager**.

Complete six degree of freedom kinematics for the tibia, talus and calcaneus were derived from fast-cine phase contrast (fast-PC or dynamic) MR images. To acquire these images, subjects were placed supine in a 1.5 T magnet (LX-9.1M4; GE Medical Systems, Milwaukee, WI, USA) with the hip and knee maintained in full extension (Figure 1). A custom-built ankle loading device (ALD [25]) supported a dual transmit-receive phased array coil medial-lateral to the foot, with the coils centered around the malleoli. The subject's sock-covered foot was strapped to the freely moving foot-pedal, which allowed three rotational degrees freedom at the plantar surface of the forefoot ("ball of the foot") and had a base plate extending to the mid-calcaneus. The ball of the foot rested on the foot pedal of the ALD. Plastic stops were placed in the ALD to limit foot-pedal rotation such that the subject's motion was maintained in a comfortable, repeatable range, typically 1-5° less than the subject's maximum calcaneal- tibial DF-PF range. The external weight system was adjusted so that a 2.3 kg (5l b) weight hung freely outside the MR imager, resulting in a resistive load being applied in calcaneal-tibial PF. The use of a cam resulted in a fixed moment arm from the rope to the center of rotation of the ALD (Figure 1). The loading level was selected as the level at which all subjects could smoothly and comfortably perform the task without reporting fatigue at the end of the trial (based on a preliminary analysis).

**Figure 2:**
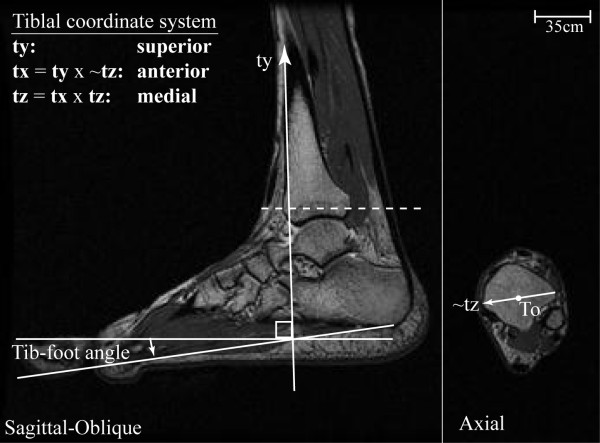
**Tibial coordinate system and tib-foot angle**.

A full fast-PC MR image set (x, y, z velocity and anatomic images over 24 time frames) was acquired while the subjects cyclically repeated a simulated single-leg toe-raises with partial-weight support for approximately 4 minutes. Subjects were asked to push the pedal down and release it back to the beat of an auditory metronome (cycle rate = 35 cycles/minute with 2 beats/cycle). This motion was not limited to PF-DF, as the three-degree of freedom pedal allowed the rear-foot joints to move in internal-external and inversion-eversion, as well. Prior to data collection, subjects practiced the task until they could comfortably repeat the motion. Axial cine images (anatomic images only) were also acquired during the movement in order to establish bone-based coordinate systems (Figure 2). Three-dimensional MR images were acquired and reviewed by a musculoskeletal radiologist to confirm the absence of foot pathology.

The IHA direction was defined as the unit angular velocity vector for each joint, expressed relative to the tibial coordinate system (Figure 2). Unlike other imaging techniques, fast-PC MRI acquires velocity data directly. Yet, the bone velocity profiles of specific anatomical points over time are not known *a priori*. Thus, the orientation and displacement of the tibia, calcaneus and talus were individually quantified by integrating velocity data obtained during the fast-PC acquisition [26]. This technique has been shown to have excellent accuracy (< 0.5 mm) [27] and subject repeatability (1.3° and 0.9 mm) [25]. From the orientation data, the direction cosine (orientation) matrices [talus relative to tibia and calcaneus relative to both talus and tibia] were defined, allowing the IHA to be quantified for all three joints [28].

All data were referenced to a tibial coordinate system, using axial and sagittal images, acquired during rear-foot supination and pronation (Figure 2), at the time frame representing the neutral tib-foot angle. The neutral tib-foot angle was defined as the point in the cycle during early calcaneal-tibial supination when the tib-foot angle was as close to zero as possible. The y-axis (**ty**) was parallel to the anterior aspect of the tibia in the sagittal image. The temporary tibial z-axis (~**tz**) was defined as the unit vector connecting the most lateral and medial tibial points on the axial image. These points were identified by finding the point at the greatest concavity on medial malleolus and on the edge of the tibia just anterior to the fibula, respectively. The final coordinate system was calculated using two cross products in order to ensure a dextral orthogonal coordinate system. The tibial origin (**To**) was defined as the point that bisected the line connecting the most lateral and medial tibial points in the axial image.

Once the orientation matrices were defined for the entire arc of motion, the angular velocity was derived for each joint of the rear-foot [29,30]:

(1) ω˜B1B2=[0−ωzωyωz0−ωx−ωyωx0]=(CB1B2)T•C˙B1B2

^B1^**ω**^B2 ^= *ω*_*x*_**t**_**x **_+ *ω*_*y*_**t**_**y **_+ *ω*_*z*_**t**_**z **_= angular velocity vector of the body B2 relative to the B1 in the B2 basis.

B1 and B2 = Body 1 and Body 2. For the talocrural joint, Body 1 = tibia and Body 2 = talus

ω_i _= angular velocity magnitude in the ith direction (i = x, y, z).

^*B*1^*C*^*B*2 ^= direction cosine matrix

^*B*1^*Ċ*^*B*2 ^= its derivative (defined using a centered finite-difference technique)

The sagittal plane point [28], defined as the point on the IHA with a medial-lateral location of zero relative to the tibial coordinate system, represented the IHA location. Since the IHA is ill-defined as ω approaches zero, data were eliminated if ω < 0.3 rad/s. Specifically, when ω = 0, the sagittal plane point is located at infinity, thus the cut-off was established so that the IHA maintained a reasonable proximity to the joint for all subjects tested.

For all analyses an orthogonal dextral coordinate system was maintained with anterior, superior and right being positive (x, y and z-directions, respectively), as recommended by the International Society of Biomechanics [31,32]. The tibial shaft-to-foot (tib-foot) angle (Figure 2) was defined to approximate the clinical ankle angle. This angle was defined as the 90° minus the angle between the vector parallel to the tibial anterior edge and the vector from the most posterior-inferior point on the calcaneus to the inferior metatarsal (typically the third metatarsal). This calculation allowed a tib-foot angle of 0° to represent the anatomical neutral position. For averaging and data presentation the z-direction along with rotations about the superior and anterior axes were negated for all right rear-feet such that medial displacement, external rotation and eversion were positive. DF was presented as a negative rotation, in order to maintain consistency with standard clinical notation. The entire movement cycle was used for all calculations, but data presentation was limited to calacaneal-tibial supination (defined as the portion of the movement with increasing tib-foot angle). Since data were taken with respect to time and not the tib-foot angle, interpolation was used to present data in single tib-foot angle increments. The translation along and the rotation about the IHA were derived post-interpolation. The range of motion a subject achieved was self-selected, as this was a volitional exercise requiring active muscle contraction. Therefore, not all subjects were represented at the extremes of the range of motion and average data points representing three or fewer subjects were eliminated from the group average.

## Results

**Figure 3:**
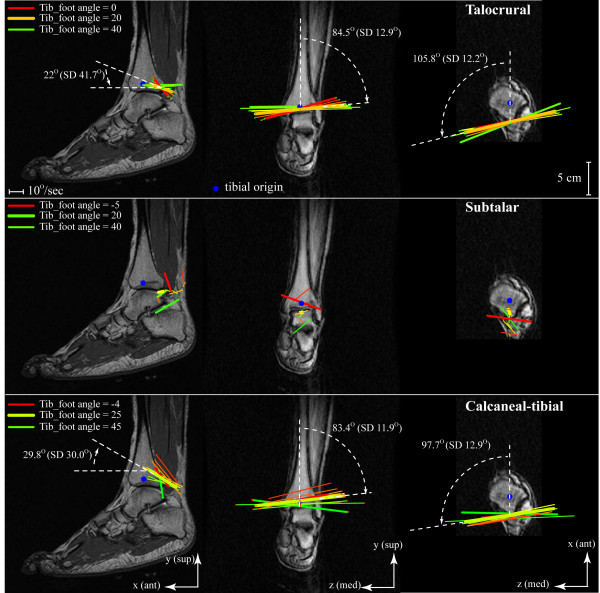
**Pictorial representation of the IHA**. For the sagittal, coronal, and axial images (left foot) the view is from lateral to medial, anterior to posterior, and distal to proximal, respectively. The maximum DF/PF is shown in the darkest shade of red/green; and the beginning, middle, and end of the PF cycle is highlighted with a thicker line. For clarity the IHA was graphed at 5° increments of tib-foot angle, instead of single degree increments. Since all images are of the same scale (280 mm^2^), the length of each IHA represents the actual angular velocity, which directly relates to the amount of rotation, at that tib-foot angle. The inclination of the IHA is provided (white dashed lines) for the talocrural and the calcaneal-tibial joints at the mid-range of motion (tib-foot angle = 20° and 25°, respectively).

**Figure 4:**
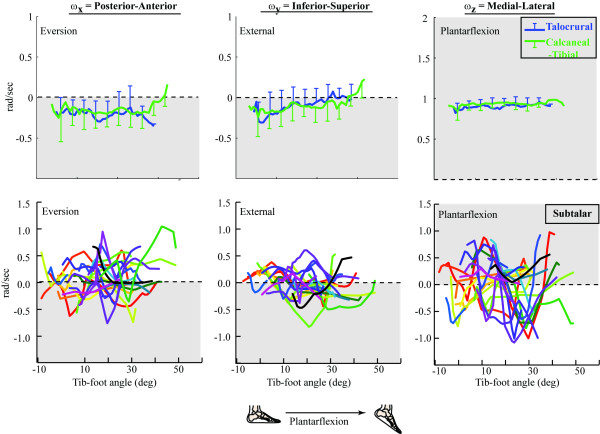
**Unit joint angular velocities**. which define the IHA direction. Pure supination occurs when all components fall within the grey areas. One SD bars are provided every 5°, except for the subtalar joint, where each subject is represented by a unique line color (due to the rapidly changing direction of the subtalar IHA, creating a subject average did not represent the data well).

The talocrural and calcaneal-tibial IHAs had similar directions, predominantly medial-lateral (Figure 3). The calcaneal-tibial displayed the expected supination pattern of PF with internal rotation and inversion (Figure 4) as did the talocrural joint. The medial and anterior directions of the IHA (indicating PF and inversion, respectively) were fairly consistent throughout the arc of motion. Yet, the axes became less inferiorly directed as the calcaneal-tibial joint supinated, (indicating diminishing internal rotation). The average direction of the subtalar IHA did not represent the kinematics well, as its direction typically changed sign in all three directions at least once during calcaneal-tibial supination for the majority of subjects (Figure 4).

The variability in the subtalar IHA resulted in the calcaneal-tibial joint having a smaller average angular velocity, relative to the talocrural joint:

$$\frac{\omega \_\text{calcaneal}-\text{tibial}}{\omega \_\text{talocrural}}=0.\text{84}\pm 0.\text{21}\left(\text{range}:0.\text{53}-\text{1}.\text{24}\right)$$

$$\frac{\omega \_\text{talocrural}}{\omega \_\text{subtalar}}=\text{2}.\text{49}\pm 0.\text{78}\left(\text{range}:\text{1}.\text{5}0-\text{4}.\text{44}\right)$$

**Figure 5:**
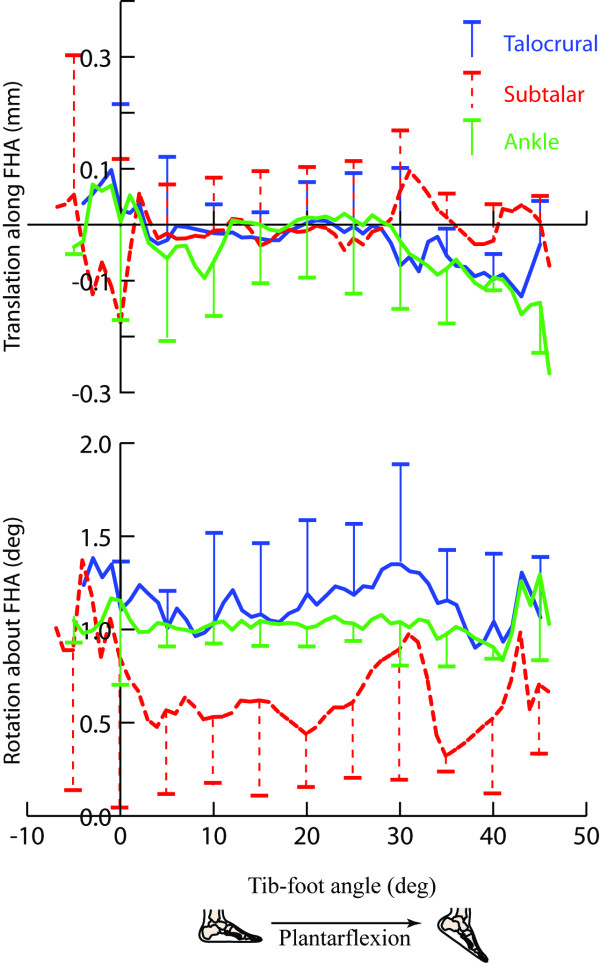
**Translation along and rotation about the IHA**. One standard deviation bars are provided every five degrees of tib-foot angle, instead of every degree increments, for clarity.

The translation along all IHAs was small and tended to be largest at extreme ranges of tib-foot angle (Figure 5). Average translations over the arc of motion were -0.5 mm (SD 1.4), -0.3 mm (SD 1.4) and -0.6 mm (SD 1.4) for the talocrural, subtalar and calcaneal-tibial joints, respectively. Total rotations about the IHA through the arc of motion, averaged across subjects, were 31.7° ± (SD 11.3°), 15.1° (SD 9.7°) and 29.1° (SD 8.5°) for the talocrural, subtalar and calcaneal-tibial joints, respectively. The variability reflects the different ranges of tib-foot angle achieved by each subject. Since rotation about the IHA is an unsigned variable, the total rotation about the IHA does not directly relate to the overall change in orientation, particularly for the subtalar joint.

**Figure 6:**
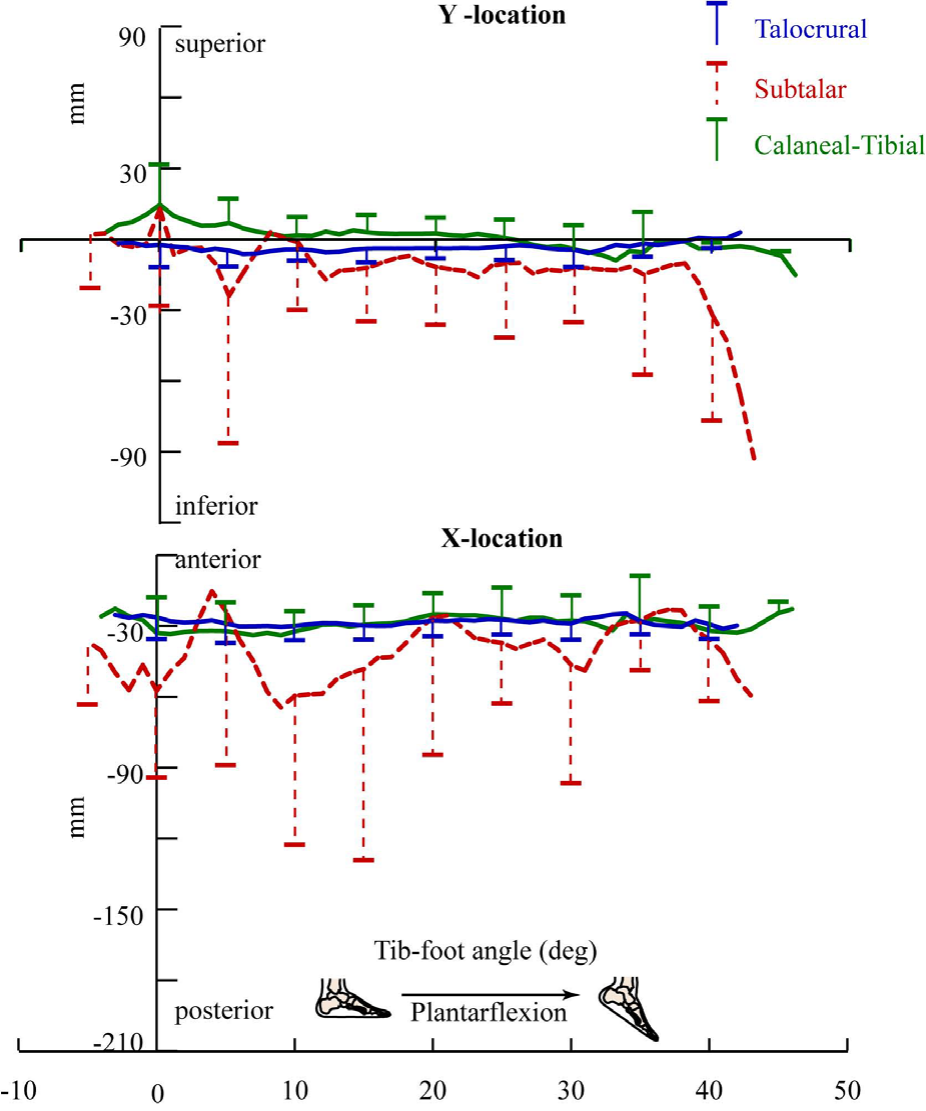
**The sagittal plane points of the IHA**. One standard deviation bars are provided every five degrees of tib-foot angle, instead of every degree, for clarity. The top and bottom graphs represent the superior-inferior and anterior-posterior location, respectively, of the sagittal plane point.

The sagittal plane point (Figure 6) was nearly identical for the talocrural and calcaneal-tibial joint throughout the range of supination. Its location varied little across the arc of motion and tended to cross the tibial origin point in the coronal plane, but remained posterior to it in the axial and sagittal planes. Adding this result to the small translation along the IHA indicates that both joints exhibit primarily rotation during calcaneal-tibial supination.

## Discussion

One of the primary findings of this study is that the IHA does not describe subtalar joint kinematics well, for the specific volitional activity examined, which agrees with the gait analysis of Arndt and colleagues [23]. The majority of the calcaneal-tibial motion was derived from the talocrural joint, with the limited subtalar rotations inconsistently opposing and supporting this motion. This highlights a primary limitation the IHA: it is a velocity measurement and is, therefore, not defined when the angular velocity approaches zero. Further, it cannot be used to define the initial pose of a joint, a major shortcoming as this is often key in defining pathology.

Arndt and colleagues [23] noted that the subtalar FHA orientation was quite variable during the gait cycle. Unfortunately, the data were presented as a single FHA over the entire gait cycle. Thus, the cycle variability was not defined. In a static *in vivo *study, Beimer and colleagues [14] found a large variation across subjects in FHA inclination (Table 1) and location (~120 mm inferior-superior range in the sagittal plane point) when measured from extreme PF to extreme DF. In addition, Lundberg and colleagues [16] demonstrated a high variability in the inclination of the subtalar FHA when quantified at different ranges of PF. Taken together, these studies support the high variability of subtalar IHA orientation and position observed across subjects and across the arc of motion. The majority of cadaver studies that quantified the FHA between maximum DF and maximum PF have shown good agreement (Table 1) with Inman's original publication [11] of a subtalar FHA inclination, with fairly low variability. This consistency is likely due to the fact that these past studies typically defined the FHA between two maximum joint positions (e.g., maximum inversion to maximum eversion or maximum PF to maximum DF). Thus, direct comparisons to these past studies are difficult and the variability reported for the subtalar IHA is likely due to the presence of active muscle control and the intact nature of the joints studies (often some or all of the soft tissue is removed during cadaver studies).

This is the first study to report complete rear-foot kinematics, based on the IHA, throughout a range of motion during a voluntary motion under active muscle control. To date, only two studies have reported *in vivo *talocrural joint kinematics [23,25], based on the FHA, during volitional activity. Arndt and colleagues [23] defined the FHA as the change in joint attitude from the beginning to the end of calcaneal-tibial PF during the gait cycle. Their limited the range of calcaneal-tibial PF (13.5°, the range of calcaneal-tibial PF is ~30° during gait [33]) was likely due to soft tissue impingement on bone pins or anesthesia. Despite these differences, the sagittal and axial plane inclination of the talocrural FHA was similar to the current results (Table 1). The rotations about the FHA were smaller in the previous study, likely due to the smaller arc of motion. In addition, the current data agree well with other previous cadaver studies in terms of the talocrural orientation (Table 1).

The translational component of motion has not been a focus of most previous studies. Yet, it is important to appreciate the small translation of the IHA and the small translation along the IHA for both the talocrural and calcaneal-tibial joints. These small translations indicate that the IHA is excellent descriptor for talocrural and calcaneal-tibial kinematics and that these joints can be modeled as fixed hinge joints. A more precise model would incorporate the small changes in IHA inclination and the small translation throughout the arc of motion.

The fact that the IHA the talocrural joint was depicted as being slightly more superior and posterior than previous reports [16] is most likely due to the motion studied. The current study focused on volitional activity requiring active muscle control, which may have allowed for greater translation of the IHA and translation along the IHA. Such a translation would maintain joint congruency with the IHA being slightly outside of the talus bone. Further, the average IHA was superimposed onto the images from a single subject. Thus, the visual interpretation relative to the bones is an approximation, as an average set of bones was not used to display the average IHA.

The primary limitation of this study was the fact that the IHA was defined for emulated partial-weight bearing, instead of full-weight bearing. This was necessary in order to exclude fatigue and maintain volunteer comfort. As open MR imaging technology improves, experiments including full-weight bearing will become available. The non-invasive dynamic nature of this experiment, its ability to incorporate muscle control and its excellent accuracy/subject-repeatability justify this potential limitation. This study is limited in its ability to define translations along the IHA, as the overall translations were within the same range as the accuracy of the technique. The variability in the subtalar IHA was not due to an inability to measure a small bone, such as the talus, with dynamic MR imaging. This is evidenced by the consistent results for the talocrural IHA, which defines the motion of the talus relative to the fairly stationary tibia, and by the excellent subject-repeatability in measuring talar motion [25]. Thus, the variability quantified is due to the true variability of the subtalar FHA both across subjects and throughout the arc of motion.

## Conclusions

The current study provides a basis for the development of improved surgical and rehabilitative protocols by establishing a normative database of rear-foot joint kinematics (represented by the IHA), acquired during a partial-weight bearing functional task. The differences between the current study and past static studies are likely due to the dynamic nature of the experiment, the required muscle activity and the inclusion of full three-dimensional rear-foot movement. The excellent subject-repeatability, high accuracy and clearly-defined coordinate systems make these data readily available for experimental comparison, modeling input and device design. Additionally, the experimental paradigm can easily be used to study impairments and the effects of intervention. In this relatively large asymptomatic population, it was obvious that the primary motion of the rear-foot (during emulated toe-raise with partial-weight support) is derived from the talocrural joint. Rotation at the subtalar joint is inconsistent and can work in both harmony and opposition to the talocrural joint in creating overall tibio-calcaneal joint movement.

## Competing interests

The author declares that they have no competing interests.
